# Association between diagnostic criteria for severe acute malnutrition and hospital mortality in children aged 6–59 months in the eastern Democratic Republic of Congo: the Lwiro cohort study

**DOI:** 10.3389/fnut.2023.1075800

**Published:** 2023-05-16

**Authors:** Gaylord Ngaboyeka, Ghislain Bisimwa, Anouk Neven, Pacifique Mwene-Batu, Richard Kambale, Petit Passy Kingwayi, Christian Chiribagula, Oreste Battisti, Michèle Dramaix, Philippe Donnen

**Affiliations:** ^1^Ecole Régionale de Santé Publique, Université Catholique de Bukavu, Bukavu, Democratic Republic of Congo; ^2^Ecole de Santé Publique, Université Libre de Bruxelles, Brussels, Belgium; ^3^Nutritional Department, Centre de Recherche en Sciences Naturelles, Lwiro, Democratic Republic of Congo; ^4^Competence Center for Methodology and Statistics, Luxembourg Institute of Health, Strassen, Luxembourg; ^5^Hôpital Provincial General de Reference de Bukavu, Université Catholique de Bukavu, Bukavu, Democratic Republic of Congo; ^6^Faculté de Médecine, Université de Kaziba, Kaziba, Democratic Republic of Congo; ^7^Département de sciences cliniques, Faculté de médecine, Université de Liège, Liège, Belgium

**Keywords:** MUAC, WHZ, MUACZ, hospital mortality, South Kivu, DRC, severe acute malnutrition

## Abstract

**Background:**

Few studies have assessed the relationship between weight-for-height (WHZ) and mid-upper arm circumference (MUAC) with hospital mortality considering confounders. The particularity of MUAC for age (MUACZ) is less documented.

**Objective:**

This study aims to investigate this relationship in a region endemic for severe acute malnutrition (SAM).

**Methods:**

This is a retrospective cohort based on a database of children admitted from 1987 to 2008 in South Kivu, eastern DRC. Our outcome was hospital mortality. To estimate the strength of the association between mortality and nutritional indices, the relative risk (RR) with its 95% confidence interval (95% CI) was calculated. In addition to univariate analyses, we constructed multivariate models from binomial regression.

**Results:**

A total of 9,969 children aged 6 to 59 months were selected with a median age of 23 months. 40.9% had SAM (according to the criteria WHZ < -3 and/or MUAC<115 mm and/or the presence of nutritional edema) including 30.2% with nutritional edema and 35.2% had both SAM and chronic malnutrition. The overall hospital mortality was 8.0% and was higher at the beginning of data collection (17.9% in 1987). In univariate analyses, children with a WHZ < -3 had a risk almost 3 times higher of dying than children without SAM. WHZ was more associated with in-hospital mortality than MUAC or MUACZ. Multivariate models confirmed the univariate results. The risk of death was also increased by the presence of edema.

**Conclusion:**

In our study, WHZ was the indicator more associated with hospital death compared with MUAC or MUACZ. As such, we recommend that all criteria shall continue to be used for admission to therapeutic SAM programs. Efforts should be encouraged to find simple tools allowing the community to accurately measure WHZ and MUACZ.

## Introduction

Acute malnutrition (AM) considerably increases the risk of morbidity and mortality in children under 5 years of age. Worldwide, 13% of deaths among these children are attributed to AM, with severe acute malnutrition (SAM) contributing to three-fifths of these deaths (1). A child with SAM has an approximately nine times greater risk of dying compared to a well-nourished child (2). It has been shown that if malnourished children are properly identified and treated, the majority of these deaths could be prevented (3). According to the World Health Organization (WHO), the identification and management of SAM is a public health priority.

Nowadays, the WHO recommends admitting children from 6 to 59 months to SAM management programs when they meet at least one of the following three criteria: (i) a *Z*-score weight for height (WHZ) < −3 relative to 2006 WHO growth standards; (ii) an Mid upper arm circumference (MUAC) < 115 mm; (iii) the presence of nutritional edema (4). Although WHZ and MUAC are recommended as independent indicators for SAM, many studies have shown that they have poor concordance (5–8).

The use of MUAC has been increasingly promoted and applied in humanitarian crisis contexts because of its simpler measurement and low cost. Indeed, screening for SAM using MUAC has been shown to be feasible by community members after a short training in remote areas where height/length boards and weighting scales are not available (8, 9). Some authors even support the exclusive use of MUAC on the basis that MUAC would be a better predictor of mortality than WHZ (8–16).

However, various studies have shown that less than 50% of the children with SAM meet both criteria (12, 17). MUAC and WHZ often identify different children and the criterion that identifies the majority of children with SAM varies considerably from country to country (12, 17).

This discrepancy in diagnosis can be partly explained by the fact that MUAC does not take age into account. MUAC for age (MUACZ) may have greater diagnostic concordance with WHZ than MUAC because of its adjustment for age. Preliminary data from Somalia do not support this hypothesis and showed that MUACZ and WHZ have low diagnostic concordance despite a similar prevalence of children with SAM (18). However, large-scale data assessing the ability of MUACZ to identify cases and predict SAM-related mortality are still scarce.

Recent analyses found that the mortality risk increases exponentially with decreasing anthropometric status measured by WHZ or MUAC and that a low WHZ has an as high or even higher risk of mortality than a low MUAC (2, 19). In addition, children with both a deficit in MUAC and WHZ have a higher mortality risk than children with a single anthropometric deficit (17, 20). According to Schwinger et al. (19) the two criteria may be the sign of different metabolic changes and therefore combining the pathological processes may potentially result in a cumulative risk of death.

As the degree of overlap between MUAC and WHZ varies greatly by context, Grellety et al. (12) recommended that both criteria continue to be used for admission to SAM programs. The two diagnostic parameters are complementary as they select different children. Failing to assess both criteria for SAM, whenever possible, would underestimate the SAM prevalence and exclude children from programs using only the opposite indicator.

In the Democratic Republic of Congo (DRC), malnutrition still remains one of the major public health problems, with a prevalence of 43% of chronic malnutrition (CM) (21). In South Kivu, a province located in the eastern DRC, malnutrition has been endemic since the 1960s (13). One in two children under the age of 5 is affected by CM and 7.9% suffers from AM with predominance of kwashiorkor (21, 22).

Data from community surveys suggest that in the DRC MUAC is the diagnostic criterion that identifies more children suffering from SAM than the WHZ criterion (12, 23–26). These studies also highlight the discordance between MUAC and WHZ in the diagnosis of SAM and the differential mortality risks for both indicators (23–26). However, all these articles are based on secondary data from nutritional surveys. The latter are often conducted in emergency contexts where the focus is mainly on MUAC. Studies evaluating the different diagnostic SAM criteria based on data collected in a medical structure specialized in the management of malnutrition in the DRC are rare. In addition, many of the published analyses assessing the survival of malnourished children have not been adjusted for important confounding factors.

The Pediatric Hospital of Lwiro (HPL) is one of the first structures that were involved in the management of malnutrition in the DRC. A team of researchers supported by the “Centre Scientifique et Médical de l’Université Libre de Bruxelles pour ses Activités de Coopération” (CEMUBAC) developed a SAM treatment model in the 1980s and has computerized the data since 1986. The electronic records contain socio-demographic, anthropometric, clinical and biological data collected from patients hospitalized between1987 and 2008, from admission to discharge from hospital.

The objective of the present study is to investigate the associations between the diagnostic indicators of SAM (WHZ, MUAC and MUACZ) and the hospital mortality of children under 5 years old treated for SAM at HPL. In our analyses, we have taken into account a number of confounding factors that may influence the survival of children, namely the presence or absence of infectious complications, that of CM and of nutritional edema.

## Materials and methods

### Study type and population

This is a retrospective cohort study based on the database of children admitted between 1987 and 2008 at the HPL for the management of SAM, in the province of South Kivu, in the DRC.

The subjects included in the present analysis were children aged 6–59 months in whom the anthropometric parameters at admission (weight, height and arm circumference), the presence of nutritional edema and of infectious disease, as well as the survival status at hospital discharge had been collected. Children with missing data for at least one of these variables have been excluded. As such, it has to be noted that children who left during hospitalization or were transferred to another facility were excluded from the study, whenever the survival status at discharge could not be retrieved. We also excluded subjects with values outside the limits defined by the WHO for *z*-scores and anthropometric parameters (23). The WHO has set the following reliability thresholds: For weight, the lower and upper limits are 3 kg and 31 kg, respectively. For height, subjects with a height < 54 cm or > 124 cm were excluded. For arm circumference, subjects with a MUAC < 75 mm or > 230 mm were excluded. For the height-for-age (HAZ) index, this concerns the subjects with a HAZ < –6 or > 6 and for the WHZ index, the subjects with a WHZ < –5 or > 5.

**Figure 1:**
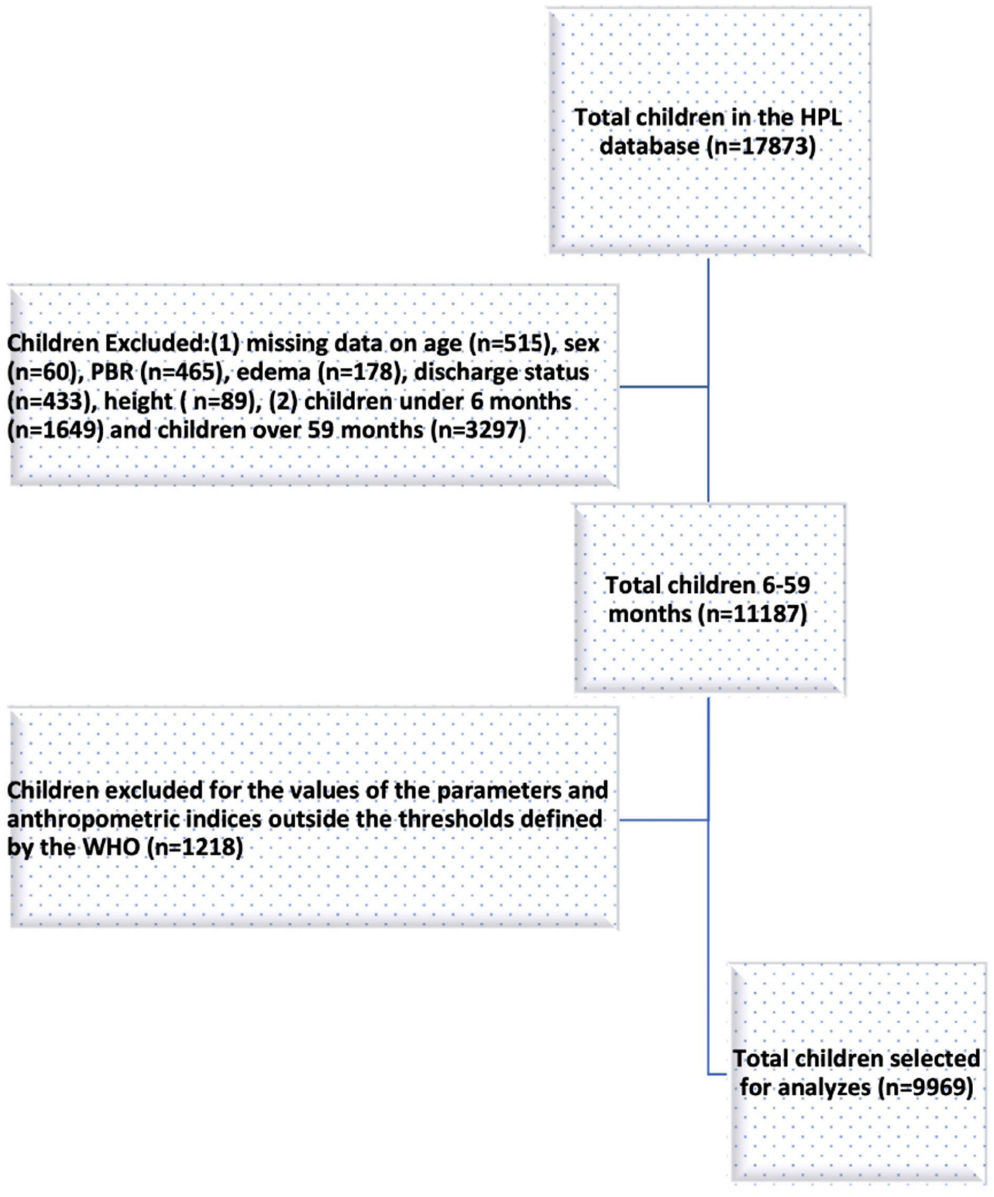
Flow chart of subjects selected for the study.

In the original HPL database from 1987 to 2008, a total of 17,873 children were recorded. Out of these children, 11,187 were aged between 6 and 59 months and had complete data for the study variables of interest. Of these, 1,218 (10.9%) additional children were excluded because at least one of their anthropometric parameters exceeded the thresholds defined by WHO, leaving 9,969 children for our analyses (Figure 1).

### Study environment

The study was conducted at the Centre de Recherche en Science Naturelle (CRSN) of Lwiro, in the health zones (HZ) of Katana and Miti-Murhesa, in South Kivu. The HZs of Miti-Murhesa and Katana are located 33 and 40 km from the city of Bukavu (capital of the province of South Kivu), respectively.

The CRSN was created in 1947 and organizes its activities in four research departments: biology, geophysics, nutrition and documentation. The nutrition department has a pediatric hospital (HPL) and several integrated health centers monitoring the health and nutrition status of children in the community.

The HPL, with a capacity of 70 beds, is located 50 km from the city of Bukavu. In the 1970s, it operated as a nutritional rehabilitation center that only admitted children suffering of SAM. Since the 1980s, it has been considered a referral hospital for all pediatric pathologies. Research activities were also carried out there. It had between 5 and 7 doctors. Consultations were provided by a general practitioner under the supervision of a pediatrician (27, 28, 41). Nutritional therapy has changed over the years, with three distinct periods. During the first period (1987–1993), treatment was based on MASOSO gruel, which is a blend of corn, soy and sorghum. A key feature in the second period (1994–1996) was the administration of locally produced high-energy milk (HEM), which was a mixture of milk, oil and sugar and had an energy density close to 900 kcal/liter. During the third period (August 1996–December 2008), HEM was replaced by the therapeutic milk F-75 (in the 1st phase of treatment) and F-100 (in the 2nd phase) (27, 28, 41).

### Study variables

The main outcome of interest was the survival status at discharge of the hospitalized child, a dichotomous variable (survivor or deceased). Hospital mortality includes death from any cause during hospitalization.

The independent variables were age (months), sex, weight (kg), height (cm), MUAC (mm), the presence or absence of nutritional edema in the hands and/or feet and/or face and the presence or absence of infection. The definition criteria and the categorization of variables are described below.

It should be noted that the nutrition diagnosis made at that time was based on the weight-for-height percentiles (WPP) of the local growth curve established by DeMaeyer in 1959 and not published (24), the presence of nutritional edema and the serum albumin level. The criteria used for diagnosis of SAM at HPL at that time differ from those currently used by the WHO.

We thus reassessed the nutritional status based on the current WHO curves (25). The anthropometric indicators (MUAC, WHZ, MUACZ, HAZ and WAZ) were each classified into three categories according to the threshold of the WHO anthropometric indicators: (1) normal, (2) moderate acute malnutrition (MAM) and (3) SAM. Children without edema whose MUAC was < 115 mm and/or WHZ *Z*-score < −3 and/or MUACZ *Z*-score < −3 were defined as having SAM.

For the MUAC and WHZ criteria, we classified children in SAM by MUAC only, by WHZ only and by both MUAC and WHZ. We have also proposed this classification for children with the MUACZ and WHZ criteria.

Children with edema were automatically classified as SAM, regardless of anthropometric indicators.

Kwashiorkor was defined by the presence of nutritional edema in the hands and/or feet and/or face and marasmus was based on a MUAC < 115 mm and/or a WHZ *Z*-score < −3 without nutritional edema. The mixed form was defined by the presence of nutritional edema with either a MUAC < 115 mm or a *Z*-score WHZ < −3 or both. In the absence of SAM, MAM was defined by a WHZ *Z*-score between −3 and − 2 and/or a MUAC between 115 and 125 mm. Finally, CM was defined by a Z-score HAZ < –2 (23).

These nutritional classifications were the ones used for the rest of the analysis.

The infectious diagnosis was based on clinical and biological assessments. The performed examinations included inflammatory assessment, direct examination of the stool, coproculture, blood culture, cytobacteriological examination of the urine, chest X-ray, lumbar puncture, analysis of the cerebrospinal fluid and thick smear.

The child age variable was categorized into three classes (6–11 months, 12–23 months, and 24–59 months).

### Ethical standards

Ethical approval was granted by the Ethics Committee of the Catholic University of Bukavu (approval number: UCB/CIE/NC/022/2016). The principle of confidentiality was respected.

### Statistical analyses

Data were analyzed with Stata.14. Frequencies and proportions were used to summarize categorical variables, the median with the interquartile range or the mean with the standard deviation were used for the description of quantitative variables depending on the shape of their distribution.

To estimate the strength of the association between the hospital mortality and the nutritional indicators, unadjusted relative risk ratios (RR) with their 95% confidence interval (95% CI) have been computed. The “normal” category has been used as reference. i.e., for MUAC the category with values ≥125 mm and for MUACZ and WHZ the category with values ≥ − 2 *Z*-score. We also reported analyses stratified by age category and presence or absence of nutritional edema.

To take into account confounding factors, adjusted relative risk ratios were calculated using multiple variable binomial regression. To deal with the collinearity between the MUAC and MUACZ indicators, two separate models were constructed. In the first model, we performed an adjustment of MUAC and WHZ indicators with age, sex, CM, presence or absence of nutritional edema and infectious diagnosis. In the second model, we performed the same adjustment by replacing MUAC with MUACZ. The chosen significance threshold was 5% (*p* < 0.05).

## Results

### General characteristics of the studied population

The children included in our study had a median age of 23 months, with almost half of them having between 24 and 59 months. In this cohort, just over half are male. In total, 30.2% of our subjects had nutritional edema and the infectious diagnosis was positive for over 80% of the children (Table 1). Of the 9,969 children admitted to the HPL during the study period, 11.5% had SAM based on WHZ only. Using MUAC only for SAM diagnosis, 14.9% suffered from SAM. Using MUACZ alone, the proportion of SAM was 21.8%. Combining WHZ and MUAC criteria for diagnosis, 36% of the children with SAM had both WHZ < –3 and MUAC < 115. When stratified by nutritional edema, 38% of SAM children with edema had a deficit in both indicators and 35% in SAM children without edema. Seventy percent of the children suffered from CM (including 48.9% suffering from the severe form). 39.1% were severely underweight (Table 1). The infectious diagnosis was made in 82.7% of children and the most frequent infections were malaria, gastroenteritis, pneumonia, and sepsis (Table 1).

**Table 1:** General characteristics of children aged 6–59 months hospitalized between 1987 and 2008 at the Lwiro Pediatric Hospital (HPL) *n* = 9,969.

Variables		% or mean (SD) or median (P25–P75)
Sex		
	Male	53.3
	Female	46.7
Age (months)		23 (12–36)^**^
	6–11	22.8
	12–23	29.1
	24–59	48.1
WHZ^***^		−1.25 (1.39)^*^
	WHZ < − 3	11.5
	3 ≤ WHZ < − 2	16.9
	2 ≤ WHZ	71.6
WAZ^***^		−2.48 (1.5)^*^
	WAZ < −3	39.1
	3 ≤ WAZ < −2	23.4
	2 ≤ WAZ	37.5
HAZ^***^		−2.84 (1.79)^*^
	HAZ < −3	48.9
	3 ≤ HAZ < −2	21.2
	2 ≤ HAZ	29.9
MUAC (mm)		131.2 (16.6)^*^
	< 115	14.9
	115 ≤ MUAC < 125	16.9
	≥ 125	68.2
MUACZ^***^		−1.81 (1.55)^*^
	MUACZ < − 3	21.8
	3 ≤ MUACZ < − 2	20.4
	2 ≤ MUACZ	57.8
Nutritional edema		
	No	69.8
	Yes	30.2
Infectious diagnosis		
	No	17.3
	Yes	82.7
	Malaria	39.9
	Gastroenteritis	13.4
	Pneumonia	8.3
	Septicemia	6.4
	Meningitis	1.3
	Skin infection (scabies)	1.3
	Measles	2
	Otitis	0.9
	Other	9.2

^*^Mean (SD); ^**^Median (P25–P75). WHZ, weight-for-height *Z*-score; MUAC, middle upper arm circumference; MUACZ, middle upper arm circumference for age *Z*-score; WAZ, weight for age *Z*-score; HAZ, height for age *Z*-score. ^***^based on the current WHO child growth curves (25).

### Malnutrition classification (based on WHO growth charts)

Based on current WHO reference curves, 40.9% of children had SAM (as criteria: nutritional edema and/or MUAC and/or WHZ). More than a third suffered from SAM and CM at the same time. Underweight concerned 62.5% of the children (Table 2).

**Table 2:** Malnutrition classification (based on WHO growth charts) of children admitted to Lwiro Pediatric Hospital (HPL).

Classification of malnutrition based on WHO curves	Numbers (%)
AM	
SAM	
Marasmus	1,064 (10.7)
Mixed	864 (8.7)
Kwashiorkor	2,145 (21.5)
MAM	1,433 (14.4)
No AM	4,463 (44.8)
CM	
Yes	6,981 (70.1)
No	2,988 (29.9)
Underweight	
Yes	6,231(62.5)
No	3,738(37.5)
Nutritional status	
SAM and CM	3,505 (35.2)
SAM only	568 (5.7)
MAM and CM	1,046 (10.5)
MAM only	387 (3.9)
CM only	2,430 (24.4)
No malnutrition	2033 (20.4)

MAM, moderate acute malnutrition; SAM, severe acute malnutrition; CM, chronic malnutrition, AM, acute malnutrition.

### Hospital mortality and its evolution over the years

**Figure 2:**
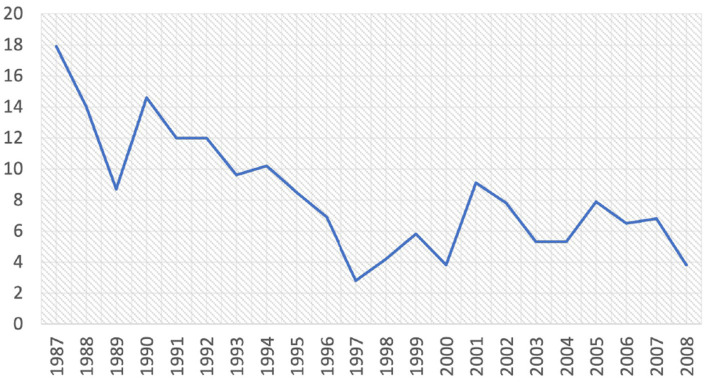
Evolution of hospital mortality over the years in children hospitalized at the Lwiro Pediatric Hospital, 1987–2008, Eastern DR Congo: Lwiro Cohort.

Over the 22 years of data collection, the average percentage of intra-hospital deaths was 8.0%. Figure 2 displays the evolution of the hospital mortality over time. At the beginning of the period mortality was highest (with 17.9% in 1987). One can observe a downward trend in mortality over time. It was 12.6% between the years 1987–1993, 8.5% between the years 1994–1996 and 5.9% between the years 1997–2008. This reduction was most probably linked to the improvement of care over the years, the improvement of diagnostic methods and the implementation of the community approach to the integrated management of acute malnutrition (PCIMA) (29–32). Efforts to improve the country’s health policies and system with the support of international partners in terms of technical and financial support could also be a factor that influenced this mortality decline over time (29, 33).

### Hospital mortality according to nutritional diagnostic criteria and characteristics (the univariate analysis)

The risk of death (compared to the “normal” category) was 2.8 times higher for children with a WHZ < –3, 2.2 times higher for SAM diagnosed by MUAC alone and 2.1 times higher for MUACZ < – 3 (Table 3); the 95% CI were largely overlapping. The risk of death was also elevated in the presence of edema and for children suffering from CM. Stratifying by age, it can be observed that the risk of death for MUAC < 115 mm or MUACZ < –3 was highest for children aged 6 to 11 months and tended to decrease with age. The risk of death for a WHZ < –3 was higher compared to the MUAC and MUACZ criteria but similar regardless of the age categories (Appendix Table 1).

**Table 3:** Hospital mortality according to nutritional diagnostic criteria and characteristics of children admitted to the HPL 1987–2008.

Variables	% of death	RR (95% CI)	*p*
Age (months)			0.753
6 to 11 (*n* = 2,273)	8.2	1.1 (0.9–1.2)	
12 to 23 (*n* = 2,897)	8.2	1.1 (0.9–1.2)	
24 to 59 (*n* = 4,799)	7.8	1	
Sex			0.003
Male (*n* = 5,310)	7.2	1	
Female (*n* = 4,659)	8.8	1.2 (1.1–1.4)	
Infection			0.784
No (*n* = 1718)	7.8	1	
Yes (*n* = 8,216)	8.0	1.0 (0.9–1.2)	
MUAC			<0.001
MUAC < 115 (*n* = 1,481)	13.8	2.2(1.9–2.6)	
115 ≤ MUAC < 125 (*n* = 1,685)	10.1	1.6(1.4–1.9)	
125 ≤ MUAC (*n* = 6,803)	6.2	1	
MUACZ			<0.001
MUACZ < −3 (*n* = 2,172)	12.5	2.1(1.8–2.5)	
−3 ≤ MUACZ < −2 (*n* = 2032)	9.3	1.6(1.3–1.9)	
−2 ≤ MUACZ (*n* = 5,765)	5.8	1	
WHZ			<0.001
WHZ < − 3 (*n* = 1,146)	16.4	2.8(2.4–3.3)	
− 3 ≤ WHZ < − 2 (*n* = 1,686)	11.3	1.9(1.6–2.3)	
− 2 ≤ WHZ (*n* = 7,137)	5.9	1	
Edema			<0.001
No (*n* = 6,960)	6.4	1	
Yes (*n* = 3,009)	11.6	1.8(1.6–2.1)	
AM			<0.001
SAM (*n* = 4,073)	11.8	2.4 (2.1–2.8)	
MAM (*n* = 1,433)	7.0	1.4 (1.1–1.8)	
No MA (*n* = 4,463)	4.8	1	
CM			<0.001
Yes (*n* = 6,981)	8.6	1.3 (1.1–1.6)	
No (*n* = 2,988)	6.5	1	

RR, relative risk; CI, confidence interval; AM, acute malnutrition; CM, chronic malnutrition; MUAC, mid-upper arm circumference; MUACZ, mid-upper arm circumference for age; WHZ, weight for height index.

### Hospital mortality according to different combinations between the criteria (WHZ, MUAC, and MUACZ)

**Figure 3:**
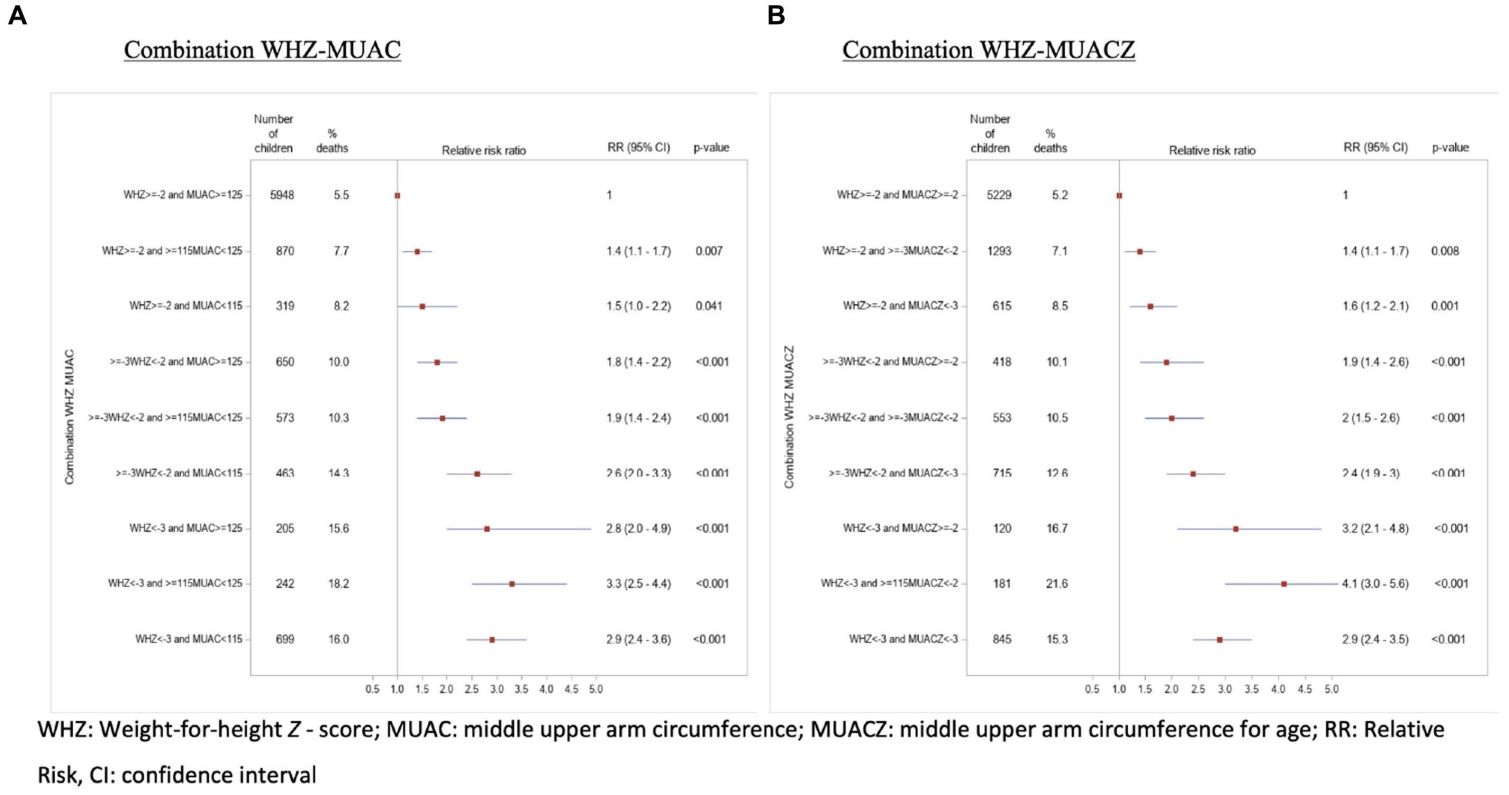
Hospital mortality according to different combinations between the weight-for-height (WHZ) and mid-upper arm circumference (MUAC) criteria on the left panel and WHZ and MUACZ on the right panel of children admitted to the HPL between 1987 and 2008.

Figure 3 shows the hospital mortality relative risk ratios for the different MUAC-WHZ and MUACZ-WHZ combinations. In the entire cohort, the risk of mortality was almost 3 times higher for children having a deficit in both criteria MUAC and WHZ or MUACZ and WHZ, respectively compared to the “normal” category. It can also be observed that the risk tended to be higher in the categories with a WHZ < − 3 than for a MUAC < 115 mm. No notable differences in the risks were observed when MUAC was replaced by MUACZ.

Regardless of the MUAC-WHZ combination, the mortality risk was systematically higher in children with edema compared to children without edema (Appendix Table 2). On the other hand, the relative risks were similar for children with and without nutritional edema. Among the children with edema, we could find a higher RR for the category “WHZ < – 3 and MUAC > = 125.” However, we cannot conclude that there was a significant difference with the other categories since the 95% confidence intervals overlap. The same observation was made when MUAC was replaced by MUACZ (Appendix Tables 2 and 3).

The reader can also find in the appendix the analyses for the combined criteria stratified by age (Appendix Tables 4 and 5).

### Hospital mortality according to nutritional diagnostic criteria and characteristics (the multivariate analysis)

The multivariate analysis (Table 4) for the WHZ and MUAC indicators showed that WHZ was most associated with mortality after adjustment for confounding factors. Indeed, children with SAM by the WHZ criterion have almost 2.4 times the risk of dying (95% CI: 1.9–2.9) compared to children without SAM. On the other hand, there was no significant association between the MUAC criterion and hospital mortality after adjustment for age, sex, presence or absence of edema, chronic malnutrition, infectious diagnosis and years of follow-up. Considering the model including MUACZ instead of MUAC, the adjusted relative risks remained similar for WHZ. Children with SAM by the MUACZ criterion were 1.2 times more likely to die than children with normal MUACZ, the difference being statistically significant. In the two adjusted models, the presence of edema increased the risk of hospital mortality by almost 2. After stratification by follow-up period (periods with changes in nutritional management), our results showed that WHZ was the criterion most associated with death compared with MUAC for all these periods. During the first two periods, the presence of edema increased the risk of death more than 4 times (Appendix Table 6).

**Table 4:** Association of nutritional indices with hospital mortality after adjustment for age, sex, presence or absence of nutritional edema, chronic malnutrition, infection and years of follow-up (binomial regression).

Nutritional indicators	Model 1 results^*^		Model 2 results^**^	
	Adjusted RR (95% CI)	*p*	Adjusted RR (95% CI)	*p*
WHZ		<0.001		<0.001
WHZ < −3	2.4(1.9–2.9)		2.3(1.9–2.8)	
−3 ≤ WHZ < −2	1.7(1.4–2.0)		1.7(1.4–2.0)	
−2 ≤ WHZ	1		1	
MUAC		0.331	Not included in model 2	
MUAC < 115	1.1 (0.9–1.3)			
115 ≤ MUAC < 125	1.1(0.9–1.4)			
125 ≤ MUAC	1			
MUACZ	Not included in model 1			0.064
MUACZ < −3			1.2(1.04–1.5)	
−3 ≤ MUACZ < −2			1.2(0.9–1.5)	
−2 ≤ MUACZ			1	
EDEMA		<0.001		<0.001
No	1		1	
Yes	2.6 (1.8–2.4)		2.0 (1.8–2.4)	

RR, relative risk; CI, confidence interval; MUAC, mid-upper arm circumference; MUACZ, mid-upper arm circumference for age; WHZ, weight for height index. ^*^Results Model 1: adjustment of MUAC and WHZ indicators with age, sex, presence or absence of nutritional edema, chronic malnutrition and infectious diagnosis. ^**^Results Model 2: adjustment of MUACZ and WHZ indicators with age, sex, presence or absence of nutritional edema, chronic malnutrition, infectious diagnosis and years of follow-up.

## Discussion

The purpose of this study was to explore the association between the WHZ, MUAC and MUACZ indicators and hospital mortality in Eastern DRC, using a database of children treated for SAM between 1988 and 2008.

Our data showed that separately, WHZ, MUAC and MUACZ are all three associated with in-hospital death. By combining the WHZ and MUAC indicators, the risk of death was highest when the child presented deficits of these two criteria concurrently. Furthermore, our univariate analysis suggested that the risk tended to be higher for children with a WHZ deficiency than for children with a low MUAC. Multivariate analysis with adjustment for age, sex, presence or absence of nutritional edema, chronic malnutrition and infection confirmed the univariate results: Children with a WHZ < – 3 were 2.5 times (95% CI: 2.0–3.1) more likely to die than children without SAM, while the association of mortality with MUAC was no longer significant after adjustment.

Our results are similar to those found by Emmanuel Grellety et al. (17), who also showed that low WHZ was more strongly associated with death than low MUAC and that the risk was increased for concurrent deficits of WHZ and MUAC. Numerous other studies have confirmed that a low WHZ alone increases significantly the risk of death (2, 34–38).

According to Katz et al. (39), the mortality risk is much higher for low WHZ in older children than in younger ones. We did not observe this difference when stratifying by age. Our results are consistent with those found by Emmanuel Grellety and Michael Golden on data from 18 African countries. Indeed, they observed that the mortality rate was higher in children with the WHZ criterion than those with the MUAC criterion for both younger and older children except for marasmus children in East Africa (17).

However, our data are not in line with the work performed by other researchers (7, 11, 14, 20) who showed that children with low WHZ were relatively healthy with a lower risk of death compared to those with low MUAC. This discordance may be partly explained by the fact that most of these studies used community data, without taking into account other confounding factors that can influence the death of malnourished children. These factors may include the presence of infections due to the defective state of immunity induced by undernutrition, CM and the presence of nutritional edema. In our study, we adjusted the risk of death for SAM diagnostic criteria by these confounding factors. Another element that can be highlighted is that in areas with a high prevalence of chronic malnutrition, children diagnosed with WHZ will be relatively few in number because most of these children will be in harmony with their weight given that they already have a short stature (chronic malnutrition). Therefore, MUAC alone will tend to recruit more SAM children. Finally, the MUAC deficit does not have the same biological explanation as the WHZ because the MUAC indicator only provides information on fat and muscle wasting whereas the WHZ provides information on all soft tissues.

In our study, we observed that a low MUACZ increased the risk of death. In the adjusted analysis, we observed that the risk of death remained significantly higher with low MUACZ as compared to children without that deficit but was lower than the risk associated with a low WHZ. Studies are rare that focus on the link between MUACZ and mortality. This would be because MUACZ is not widely used in many countries. Studies to shed light on this are to be encouraged.

We agree with other researcher that the use of MUAC is relatively simple, inexpensive, and therefore facilitates community screening for SAM. However, its sole use would lead to the non-identification of a significant proportion of children suffering from SAM who do not have a low MUAC but rather a low MUACZ and/or a low WHZ. Given that the objective of community management programs for SAM is to recruit as many affected children as possible and to reduce SAM-related mortality, the use of all anthropometric criteria (WHZ, MUAC, MUACZ) should be encouraged. It is therefore necessary to develop sufficiently simple tools allowing to measure WHZ and/or MUACZ in the community, so that children with WHZ < –3 and MUACZ < –3 can also be included in care programs. With modern technology, the use of smart phones connected to scanning rooms would give accurate measurements of height, head circumference and MUAC (7, 12, 15, 16). With these advances, we hope to have techniques available in the future that make the assessment of WHZ and MUACZ simple to use in community settings. While waiting for these scientific advances, intensive care units for SAM must take into account all diagnostic criteria for admission to therapeutic SAM programs, whenever possible. We also encourage studies aimed at defining a threshold of MUAC which could potentially identify all SAM children by MUACZ and WHZ in the community and also a clinical score in relation to the clinical signs providing information on nutrient deficiencies and thus allow easy identification in the community of all SAM children and triage for management in a medical structure where all criteria can be easily used. Specific nutrient deficiencies have not been studied for children followed for SAM because we did not have information on their dosage. Nutrient deficiencies will be very important to know to better care for children and also reduce the risk of mortality.

Another finding observed is that the overall hospital mortality was 8.0% during the study period and was highest at the start of collection. This could be the results of the efforts made over the years, from the evolution of screening over the management of malnutrition.

Finally, children with nutritional edema are suffering from SAM according to the WHO definitions. The latter are at approximately twice the risk of death in our study compared to children without edema. This is explained by the fact that SAM reduces the immune system and thus exposes to severe infections and many other acute complications.

In our study, we found that more than 80% of children followed at the HPL had an infection. Although the HPL worked as a nutritional center, it also took care of other children’s illnesses given the need of the population and the accessibility to the center, which could explain the high prevalence of infections in our dataset. A second explanation for this proportion of infections would be SAM itself with the drop-in immunity it induces, thus explaining exposure to infections.

Despite our results, some limitations should be mentioned. First, information about the psychomotor development of each child from birth is not known. Moreover, the notion of relapse episodes of malnutrition for each child given the endemic context of AM in the study region, the duration of hospitalization and the secondary causes of AM are not known. These items could be potential confounding factors, as they are linked to the survival of children in hospitalization. Moreover, certain records were excluded at the beginning of the study because the subjects had incomplete data, left during hospitalization or were transferred to another facility. These children for whom we have no information could have left the hospital and died elsewhere of various causes, including cases of relapses, but also cases of many other complications. Consequently, we do not know how the data on these subjects would have influenced our results, but the number is small, we do not expect the results to change significantly. Another limitation is the absence of HIV serology in our analysis due to its association with malnutrition and mortality. Nevertheless, two recent studies in our environment, including one on the cohort of adults with a history of SAM, show a low prevalence of HIV in the malnourished. In view of this, we believe that this would not have substantially influenced our main conclusions (28, 39). Finally, the absence of information on the duration of hospitalization of each child did not allow us to perform a Cox regression analysis which is a standard method to study time-to-event endpoints.

Despite these limitations, our study is one of the few research projects that investigates the association between hospital mortality and different anthropometric indicators in such a large cohort of children (nearly 10,000 children) followed in a tertiary level structure, while taking into account certain clinical aspects.

In conclusion, our work has shown that WHZ alone is more strongly associated with hospital mortality than MUAC or MUACZ alone after adjustment for age, sex, the presence of nutritional edema, infection, and chronic malnutrition. The presence of nutritional edema also increases the risk of death in our environment and presents an additive effect of death to the other indicators of SAM. As such, we think that all criteria shall be used for admission to therapeutic SAM programs. Efforts should be encouraged to find simple tools allowing the community to accurately measure WHZ and MUACZ, so that children with WHZ < –3 and/or MUACZ < – 3 can also be included in prevention programs. As for example, Buonomo et al. who show in their study that the assessment of weight velocity *Z*-scores, coupled with the already validated malnutrition indices, can support frontline health workers in early prediction of child malnutrition (40).

## Data availability statement

The datasets presented in this study can be found in online repositories. The names of the repository/repositories and accession number(s) can be found in the article/Supplementary material.

## Ethics statement

The studies involving human participants were reviewed and approved by Université catholique de Bukavu. Written informed consent to participate in this study was provided by the participants’ legal guardian/next of kin. Written informed consent was obtained from the individual(s), and minor(s)' legal guardian/next of kin, for the publication of any potentially identifiable images or data included in this article.

## Author contributions

PD, GB, MD, and GN designed the study. MD, AN, and GN analyzed the data and produced tables. GN wrote the manuscript. PD, MD, GB, OB, AN, PM-B, RK, CC, PP, and GN critically reviewed the manuscript for its significant intellectual content. PD, MD, GB, PM-B, RK, OB, AN, and GN read and approved the final manuscript. All authors have read and approved the manuscript, have full access to all data, and take responsibility for the integrity of the data and the accuracy of the data analysis.

## Funding

This study was supported by the Research Center “Politics and systems of international health-health” of the Université Libre de Bruxelles.

## Conflict of interest

The authors declare that the research was conducted in the absence of any commercial or financial relationships that could be construed as a potential conflict of interest.

## Publisher’s note

All claims expressed in this article are solely those of the authors and do not necessarily represent those of their affiliated organizations, or those of the publisher, the editors and the reviewers. Any product that may be evaluated in this article, or claim that may be made by its manufacturer, is not guaranteed or endorsed by the publisher.

## Abbreviations

HPL: Lwiro Pediatric Hospital
MAS: severe acute malnutrition
WHO: World Health Organization
PCMA: community management of acute malnutrition
DRC: Democratic Republic of Congo
WHZ: weight-for-height *Z*-score
MUAC: middle upper arm circumference
MUACZ: middle upper arm circumference for age *Z*-score
CEMUBAC: Centre Scientifique et Médical de l’Université Libre de Bruxelles pour ses Activités de Coopération
HZ: health zone
AM: acute malnutrition
WAZ: weight for age *Z*-score
HAZ: height for age *Z*-score
CRSN-Lwiro: Centre de Recherche en Sciences Naturelles de Lwiro
NCHS: National Center for Health Statistics
CM: chronic malnutrition
DRC: Democratic Republic of Congo
