# Supplementary material for: Association between diagnostic criteria for severe acute malnutrition and hospital mortality in children aged 6–59 months in the eastern Democratic Republic of Congo: the Lwiro cohort study
Source: Front Nutr. 2023 May 16;10:1075800. doi: 10.3389/fnut.2023.1075800 (PMC10246449; doi:10.3389/fnut.2023.1075800)
Supplement: Supplementary file 1 [file Data_Sheet_1.zip › Appendix Table 1.pdf]

Table Appendix 1. Association between nutritional diagnostic criterion and risk of hospital mortality during the study period stratified by age category of children admitted to the HPL between 1987 and 2008

	6-11 months				12-23 months				24-59 months			
MUAC	n	% Death	RR (95% CI)	P	n	% Death	RR (95% CI)	P	n	% Death	RR (95% CI)	P
MUAC<115	423	14.90%	2.58(1.89-3.52)	<0.001	539	13.90%	2.27(1.72-2.99)	<0.001	519	12.70%	1.98(1.53-2.57)	<0.001
115≤MUAC<125	447	9.40%	1.63(1.14-2.33)	0.008	498	9.60%	1.57(1.14-2.17)	0.006	740	10.80%	1.69(1.32-2.15)	<0.001
125 ≤ MUAC	1403	5,80%	1		1860	6.10%	1		3540	6.40%	1	
WHZ												
WHZ < −3	239	15.90%	2.85(2.01-4.06)	<0.001	383	17.00%	2.96(2.23-3.94)	<0.001	524	16.20%	2.67(2.11-3.38)	<0.001
−3 ≤ WHZ < −2	365	15.10%	2.7 (1.97-3.70)	<0.001	523	11.10%	1.94 (1.43-2.62)	<0.001	798	9.60%	1.59 (1.24-2.04)	<0.001
−2 ≤ WHZ	1669	5.60%	1		1991	5.70%	1		3477	6.10%	1	
MUACZ												
MUACZ <−3	363	14.30%	2.51 (1.81-3.46)	<0.001	590	13.20%	2.19 (1.66-2.89)	<0.001	1222	11.50%	2.01 (1.61-2.51)	<0.001
−3 ≤ MUACZ <−2	388	12.10%	2.12 (1.51-2.97)	<0.001	532	9.80%	1.62 (1.18-2.23)	0.003	1107	8.10%	1.41 (1.09-1.82)	0.007
−2 ≤ MUACZ	1522	5.70%	1		1775	6.00%	1		2470	5.70%	1	

WHZ: Weight-for-height Z - score; MUAC: middle upper arm circumference; MUACZ: middle upper arm circumference for age; RR: Relative Risk; CI: confidence interval
