# Supplementary material for: Association between diagnostic criteria for severe acute malnutrition and hospital mortality in children aged 6–59 months in the eastern Democratic Republic of Congo: the Lwiro cohort study
Source: Front Nutr. 2023 May 16;10:1075800. doi: 10.3389/fnut.2023.1075800 (PMC10246449; doi:10.3389/fnut.2023.1075800)
Supplement: Supplementary file 1 [file Data_Sheet_1.zip › Appendix Table 2.pdf]

Table Appendix 2. Hospital mortality according to different combinations between the WHZ and MUAC criteria of children admitted to the HPL stratified by the presence or absence of nutritional edema between 1987 and 2008

Combination MUAC and WHZ	Whole group				Presence of nutritional edema				Absence of nutritional edema			
	n	Death %	RR(95% CI)	P*	n	Death %	RR (95% CI)	P*	n	Death %	RR (95% CI)	P*
WHZ<-3 and MUAC<115	699	16,0	2.9 (2.4-3.6)	<0,001	325	17,2	2.3(1.7-3.2)	<0,001	374	15,0	3.1(2.3-4.1)	<0,001
WHZ<-3 and >=115MUAC<125	242	18,2	3.3 (2.5-4.4)	<0,001	89	23,6	3.2(2.1-4.8)	<0,001	153	15,0	3.1(2.1-4.6)	<0,001
WHZ<-3 and MUAC≥125	205	15,6	2.8 (2.0-4.9)	<0,001	54	35,2	4.8(3.2-7.2)	<0,001	151	8,6	1.8(1.0-3.0)	0,035
>=-3WHZ<-2 and MUAC<115	463	14,3	2.6 (2.0-3.3)	<0,001	245	16,3	2.2(1.6-3.1)	<0,001	218	11,9	2.5(1.7-3.6)	<0,001
>=-3WHZ<-2 and >=115MUAC<125	573	10,3	1.9 (1.4-2.4)	<0,001	198	13,1	1.8(1.2-2.7)	0,005	375	8,8	1.8(1.3-2.6)	0,001
>=-3WHZ<-2 and MUAC≥125	650	10,0	1.8 (1.4-2.2)	<0,001	156	19,2	2.6(1.8-3.8)	<0,001	494	7,1	1.5(1.0-2.1)	0,030
WHZ>=-2 and MUAC<115	319	8,2	1.5 (1.0-2.2)	0,041	151	9,3	1.3(0.7-2.1)	0,388	168	7,1	1.5(0.8-2.6)	0,174
WHZ>=-2 and >=115MUAC<125	870	7,7	1.4 (1.1-1.7)	0,007	306	11,4	1.5(1.1-2.2)	0,016	564	5,7	1.2(0.8-1.7)	0,388
WHZ>=-2 and MUAC≥125	5948	5,5	1		1485	7,3	1		4463	4.8	1	

WHZ: Weight-for-height Z - score; MUAC: middle upper arm circumference; RR: Relative Risk; CI: confidence interval
