# Supplementary material for: Association between diagnostic criteria for severe acute malnutrition and hospital mortality in children aged 6–59 months in the eastern Democratic Republic of Congo: the Lwiro cohort study
Source: Front Nutr. 2023 May 16;10:1075800. doi: 10.3389/fnut.2023.1075800 (PMC10246449; doi:10.3389/fnut.2023.1075800)
Supplement: Supplementary file 1 [file Data_Sheet_1.zip › Appendix Table 3.pdf]

Appendix Table 3. Hospital mortality according to different combinations between the WHZ and MUACZ criteria of children admitted to the HPL stratified by the presence or absence of nutritional edema between 1987 and 2008

Combination MUACZ and WHZ	Presence of nutritional edema				Absence of nutritional edema			
	n	Death %	RR (95% CI)	P*	n	Death %	RR(IC95%)	P*
WHZ<-3 and MUACZ<-3	392	17,6	2.6(1.9-3.6)	<0.001	453	13,25	2.7(2.1-3.6)	<0.001
WHZ<-3 and >=115MUACZ<-2	55	32,73	4.9(3.1-7.5)	<0.001	126	16,67	3.4(2.3-5.2)	<0.001
WHZ<-3 and MUACZ≥-2	21	42,86	6.4(3.7-10.9)	<0.001	99	11,11	2.3(1.3-4.1)	0.004
>=-3WHZ<-2 and MUACZ<-3	373	13,94	2.1(1.5-2.9)	<0.001	342	11,11	2.3(1.6-3.2)	<0.001
>=-3WHZ<-2 and >=-3MUACZ<-2	155	18,06	2.7(1.8-4.0)	<0.001	398	7,54	1.6(1.1-2.2)	0.019
>=-3WHZ<-2 and MUACZ≥-2	71	22,54	3.3(2.1-5.4)	<0.001	347	7,49	1.5(1.0-2.3)	0.030
WHZ>=-2 and MUACZ<-3	351	11,4	1.7(1.2-2.4)	0.005	264	4,55	0.9(0.5-1.7)	0.823
WHZ>=-2 and >=-3MUACZ<-2	506	8,89	1.3(0.9-1.9)	0,125	787	5,97	1.2(0.9-1.7)	0.186
WHZ>=-2 and MUACZ ≥-2	1085	6.73	1		4,144	4.85	1	

WHZ: Weight-for-height Z - score; MUACZ: middle upper arm circumference for age; RR: Relative Risk; CI: confidence interval
