# Supplementary material for: Association between diagnostic criteria for severe acute malnutrition and hospital mortality in children aged 6–59 months in the eastern Democratic Republic of Congo: the Lwiro cohort study
Source: Front Nutr. 2023 May 16;10:1075800. doi: 10.3389/fnut.2023.1075800 (PMC10246449; doi:10.3389/fnut.2023.1075800)
Supplement: Supplementary file 1 [file Data_Sheet_1.zip › Appendix Table 4.pdf]

Appendix Table 4. Hospital mortality for the different combinations between the WHZ and MUAC criteria during the study period stratified by age category between 1987 and 2008

Combination MUAC and WHZ	6-11 months				12-23 months				24-59 months			
	n	% Death	RR (95% CI)	P	n	% Death	RR (95% CI)	P	n	% Death	RR (95% CI)	P
WHZ<-3 and MUAC<115	176	16.48	3.25(2.16-4.88)	<0.001	271	14.76	2.79(1.96-3.96)	<0.001	252	17.06	2.98(2.19-4.06)	<0.001
WHZ<-3 and >=115MUAC<125	31	16.13	3.18(1.38-7.35)	0.006	69	24.64	4.66(2.94-7.38)	<0.001	142	15.49	2.71(1.79-4.08)	<0.001
WHZ<-3 and MUAC≥125	32	12.5	2.47(0.96-6.35)	0.062	43	18.6	3.52(1.82-6.77)	0.0002	130	15.38	2.69(1.75-4.12)	<0.001
>=-3WHZ<-2 and MUAC<115	133	10.8	3.71(2.42-5.67)	<0.001	173	13.87	2.62(1.72-4.005)	<0.001	157	10.83	1.89(1.18-3.03)	0.008
>=-3WHZ<-2 and >=115MUAC<125	143	12.59	2.48(1.52-4.06)	0.0003	177	8.47	1.60(0.95-2.71)	0.079	253	10.28	1.79(1.21-2.66)	0.003
>=-3WHZ<-2 and MUAC≥125	89	13.48	2.66(1.49-4.74)	0.0009	173	10.98	2.08(1.29-3.32)	0.0024	388	8.76	1.53(1.08-2.18)	0.017
WHZ>=-2 and MUAC<115	114	7.89	1.56(0.79-3.04)	0.1954	95	11.58	2.19(1.21-3.96)	0.0095	110	5.45	0.95(0.43-2.10)	0.905
WHZ>=-2 and >=115MUAC<125	273	6.96	1.37(0.84-2.25)	0.201	252	6.35	1.19(0.72-2.01)	0.4894	345	9.28	1.62(1.13-2.32)	0.009
WHZ>=-2 and MUAC≥125	1282	5.07	1		1644	5.29	1		3022	5.72	1	

WHZ: Weight-for-height *Z*-score; MUAC: middle upper arm circumference; RR: relative risk ratio; CI: confidence interval
