# Supplementary material for: Association between diagnostic criteria for severe acute malnutrition and hospital mortality in children aged 6–59 months in the eastern Democratic Republic of Congo: the Lwiro cohort study
Source: Front Nutr. 2023 May 16;10:1075800. doi: 10.3389/fnut.2023.1075800 (PMC10246449; doi:10.3389/fnut.2023.1075800)
Supplement: Supplementary file 1 [file Data_Sheet_1.zip › Appendix Table 5.pdf]

**Appendix Table 5. Hospital mortality for the different combinations between the WHZ and MUACZ criteria during the study period stratified by age category between 1987 and 2008**

| Combination MUACZ and WHZ | 6-11 months | | | | 12-23 months | | | | 24-59 months | | | |
|---|---|---|---|---|---|---|---|---|---|---|---|---|
| | n | % Death | RR (95% CI) | P | n | % Death | RR (95% CI) | P | n | % Death | RR (95% CI) | P |
| WHZ<-3 and MUACZ<-3 | 164 | 15.24 | 3.08(2.01-4.73) | <0.0001 | 285 | 14.74 | 2.88(2.03-4.09) | <0.0001 | 396 | 15.66 | 2.85(2.14-3.78) | <0.0001 |
| WHZ<-3 and >=115MUACZ<-2 | 40 | 20.00 | 4.09(2.09-7.84) | <0.0001 | 58 | 25.86 | 5.05(3.11-8.20) | <0.0001 | 83 | 19.28 | 3.50(2.19-5.62) | <0.0001 |
| WHZ<-3 and MUACZ≥-2 | 35 | 14.29 | 2.89(1.24-6.72) | 0.0138 | 40 | 20.00 | 3.91(2.03-7.52) | <0.0001 | 45 | 15.56 | 2.83(1.40-5.70) | 0.0037 |
| >=-3WHZ<-2 and MUACZ<-3 | 118 | 18.64 | 3.77(2.42-5.87) | <0.0001 | 199 | 12.06 | 2.36(1.53-3.62) | 0.0001 | 398 | 11.06 | 2.01(1.45-2.79) | <0.0001 |
| >=-3WHZ<-2 and >=-3MUACZ<-2 | 135 | 14.07 | 2.85(1.77-4.58) | <0.0001 | 171 | 9.36 | 1.83(1.09-3.05) | 0.0211 | 247 | 9.31 | 1.69(1.11-2.59) | 0.0152 |
| >=-3WHZ<-2 and MUACZ≥-2 | 112 | 12.50 | 2.52(1.47-4.35) | 0.0008 | 153 | 11.76 | 2.30(1.42-3.72) | 0.0007 | 153 | 6.54 | 1.19(0.64-2.21) | 0.5879 |
| WHZ>=-2 and MUACZ<-3 | 81 | 6.17 | 1.25(0.52-3.01) | 0.6215 | 106 | 11.32 | 2.21(1.25-3.92) | 0.0067 | 428 | 8.18 | 1.49(1.04-2.13) | 0.0311 |
| WHZ>=-2 and >=-3MUACZ<-2 | 213 | 9.39 | 1.89(1.18-3.06) | 0.0085 | 303 | 6.93 | 1.35(0.85-2.15) | 0.2008 | 777 | 6.56 | 1.19(0.87-1.64) | 0.2726 |
| WHZ>=-2 and MUACZ≥-2 | 1375 | 4.95 | 1 | | 1,582 | 5.12 | 1 | | 2272 | 5.50 | 1 | |

WHZ: Weight-for-height Z - score; MUACZ: middle upper arm circumference for age; RR: Relative Risk; CI: confidence interval
