# Supplementary material for: Association between diagnostic criteria for severe acute malnutrition and hospital mortality in children aged 6–59 months in the eastern Democratic Republic of Congo: the Lwiro cohort study
Source: Front Nutr. 2023 May 16;10:1075800. doi: 10.3389/fnut.2023.1075800 (PMC10246449; doi:10.3389/fnut.2023.1075800)
Supplement: Supplementary file 1 [file Data_Sheet_1.zip › Appendix Table 6.pdf]

Appendix Table 6. Association of nutritional indices with hospital mortality after adjustment for age, sex, presence or absence of nutritional edema, chronic malnutrition, and infection stratified by follow-up periods (Binomial regression)

Model 1*	Follow-up Period 1987-1993		Follow-up Period 1994-1996		Follow-up Period 1997-2008	
	RRa (95% CI)	p	RRa (95% CI)	p	RRa (95% CI)	p
WHZ		<0.001		0.003		<0.001
WHZ < −3	3.2(2.2-4.7)		2.9(1.4-5.9)		2.2(1.5-3.0)	
−3 ≤ WHZ < −2	2.3(1.7-3.3)		1.5(0.8-2.8)		1.5(1.1-2.0)	
−2 ≤ WHZ	1		1		1	
MUAC		0.102		0.044		0.361
MUAC < 115	1.4(1.0-1.9)		2.2(1.1-4.5)		1.2(0.9-1.6)	
115≤MUAC<125	1.3(0.9-2.0)		1.1(0.5-2.1)		1.2(0.8-1.6)	
125 ≤ MUAC	1				1	
EDEMA		<0.001		<0.001		0.248
No	1		1		1	
Yes	5.3(3.9-7.1)		4.1(2.4-6.8)		1.2(0.9-1.5)	
Model 2**						
WHZ		<0.001		0.002		<0.001
WHZ < −3	3.1(2.1-4.6)		3.0(1.5-6.1)		2.1(1.5-2.9)	
−3 ≤ WHZ < −2	2.2(1.6-3.1)		1.5(0.8-2.8)		1.5(1.1-2.0)	
−2 ≤ WHZ	1		1		1	
MUACZ		0.040		0.064		0.191
MUACZ< −3	1.6(1.1-2.2)		2.0(0.9-4.1)		1.2(0.9-1.6)	
−3 ≤ MUACZ < −2	1.4(0.9-2.0)		0.9(0.5-1.9)		1.2(0.9-1.7)	
−2 ≤ MUACZ	1		1		1	
EDEMA		<0.001		<0.001		0.285
No	1		1		1	
Yes	5.2(3.9-7.0)		4.2(2.5-7.0)		1.1(0.9-1.5)	

RR: Relative Risk; CI= Confidence interval; MUAC: Mid-upper arm circumference; MUACZ: Mid-upper arm circumference for age; WHZ: Weight for height index; *Results Model 1: Adjustment of MUAC and WHZ indicators with age, sex, presence or absence of nutritional edema,

chronic malnutrition and infectious diagnosis ** Results Model 2: Adjustment of MUACZ and WHZ indicators with age, sex, presence or absence of nutritional edema, chronic malnutrition and infectious diagnosis
